# Out of hospital cardiac arrest: when to resuscitate

**DOI:** 10.11604/pamj.2019.33.289.17583

**Published:** 2019-08-07

**Authors:** Saida Zelfani, Hela Manai, Yosra Riahi, Mounir Daghfous

**Affiliations:** 1 Pre-Hospital Emergency Department (SAMU 01), Emergency Medical Help Center of Tunis, Tunis, Tunisia

**Keywords:** Out of hospital cardiac arrest, cardiopulmonary resuscitation, pre hospital emergency services

## Abstract

**Introduction:**

This study explores why resuscitation is withheld when mobile emergency medical team arrive at the scene of a cardiac arrest.

**Methods:**

We conducted a prospective, observational study in pre hospital emergency services. We included adults' patients, with a suspicion of non-traumatic cardiac arrest (CA) in an out of hospital environment, who received or not cardiopulmonary resuscitation (CPR) by our mobile emergency medical service teams. An analytic study was conducted in order to identify independent factors that could influence the decision to resuscitate OHCA.

**Results:**

During study, 228 patients were enrolled, the mean age was 64 +/- 14 years and 59% were men. Eighteen patients (8%) received bystander CPR by witnesses. The median time elapsed to arrive at the scene was 13 [8-25] min. The median “noflow” was 22 [10-34] min. The resuscitation decision was taken by the mobile EMS staff for 106 patients (46.5%). For other patients, the decision not to resuscitate was motivated solely by the finding of a confirmed state of death in an elderly patient (p = 0.045). The predictive decision factor for resuscitation was the no flow time less than 18.5 min, Odds Ratio adjusted with 95% confidence interval to: 1.38 (1.24 - 3.55) (p <0.001). Overall out of hospital survival rate was 17% of resuscitated patients.

**Conclusion:**

The decision to resuscitate a cardiac arrest outside of the hospital depends more on the “no flow” time than on the presumed etiologies.

## Introduction

When cardiac arrest (CA) occurs there is sudden cessation of circulation to the brain and other vital organs. Irreversible death will occur within minutes unless circulation is restored. The technique of cardiopulmonary resuscitation (CPR) can be used to buy time whilst reversible causes of cardiac arrest are identified and treated [1]. Data indicate that CPR is only initiated or continued by mobile emergency medical service (EMS) in approximately 28,000 cases. This suggests that in more than 50%of cardiac arrests, resuscitation is withheld by mobile EMS [2]. Most out of hospital cardiac arrest (OHCA) occur in the absence of healthcare staff. Here, bystander CPR can improve survival chances by two to four folds [3,4]. Despite its lifesaving potential, circumstances exist where attempting resuscitation is inappropriate. This includes un-survivable injuries or clear evidence of death (e.g. rigor mortis, post mortem staining). Resuscitation is also withheld by mobile EMS teams when there is no prospect of success. Little is known about the characteristics of patients in whom resuscitation is withheld by mobile EMS. This study aimed to determine the reasons for resuscitating or not an OHCA by the mobile EMS teams when they arrived at the scene.

## Methods

**Study population:** we conducted a prospective observational study over a period of two years (January 2015 to December 2016) in the pre hospital emergency department, north east of Tunisia. We included all adults patients aged more than 18 years old, with a suspicion of non-traumatic CA in an out of hospital environment, who received or not CPR by our mobile EMS teams. We excluded secondarily patients with other than CA diagnosis.

**Group comparison:** to study the factors that could influence the decision to resuscitate OHCA, we divided the total population into two subgroups: group resuscitation+: patients received advanced life support by mobile EMS teams; group resuscitation: patients not received advanced life support by mobile EMS teams.

**Data collection:** information collected detailed patients characteristics (age, sex), Comorbidities, witnesses (doctors, paramedics, rescuers, other), bystander CPR (present/absent), no flow time, ambulance response time, initial electrocardiographic rhythm, whether ambulance staff attempted resuscitation or not, survival rate.

**Out of hospital protocol:** upon confirmation of CA, mobile EMS assessed the appropriateness of initiating resuscitation (or if it was bystander initiated, to continue it). If resuscitation was appropriate, it was initiated according to standardized guidelines based on European Resuscitation Council guidelines 2015. Patients were then transported to an emergency department (with return of spontaneous circulation or ongoing CPR) or declared deceased if non reversible causes of CA were identified. As it may take a few minutes after mobile EMS arrival to establish if resuscitation is appropriate, short resuscitation attempts (where mobile EMS resuscitation duration was under 3 minutes) were categorized as non-resuscitation attempts. If mobile EMS clinicians are presented with signs unequivocally associated with death or resuscitation was deemed futile, they did not provide CPR.

**Statistical analysis:** we used SPSS, version 20.0 (IBM SPSS Inc, Chicago, Illinois, USA) for data analysis. The Kolmogorov-Smirnov test as used for variables distribution. Categorical values were assessed using a chi-square test (or Fisher's exact test when indicated) and continuous variables using a Student T test or Mann-Whitney test for trends in the absence of a normal distribution. Analysis was performed with logistic regression by backward stepwise elimination. The odds ratio (OR) was expressed with the respective 95% CI. In all tests, a p-value less than 0.05 was significant. Univariate analysis of baseline variables was performed by using a backward stepwise variable selection procedure to determine the predictive factors of the decision to resuscitate OHCA. To study the independent predictors of cardiac arrest resuscitation, multivariate.

## Results

**Figure 1:**
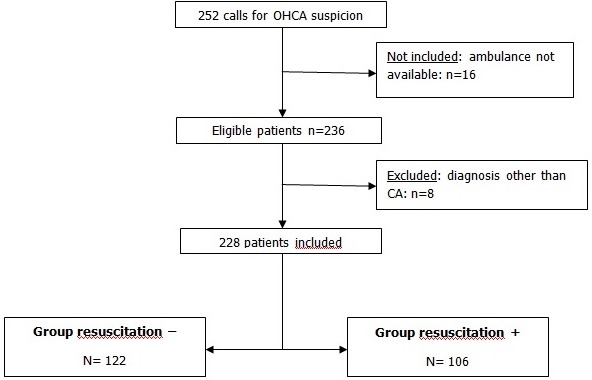
Inclusion algorithm

**Baseline characteristics of the study population:** during the study period, there were 252 calls for non-traumatic OHCA. We didn't include 16 patients because they were not treated by our mobile EMS teams. There were 236 patients eligible for inclusion; however 8 patients were excluded because of the diagnosis established by the mobile EMS doctors is not a cardiac arrest. A total of 228 patients (5% of all our interventions) were enrolled into the study (Figure 1). Mean age was 64±14 years. Ninety nine patients (43%) were aged more than 65 years. Males were predominant (59%) with a sex-ratio=1.42. The median time of ambulance departure was 6 min (4-10) whereas the median time elapsed to arrive at the scene was 13 min (8-25). Before the arrival of the ambulance 18 patients (8%) received bystander CPR by witnesses (2 rescuers, 4 nurses and 12 doctors). Of these, two patients have recovered cardiac activity. Sixteen OHCA occurred at the arrival of the mobile EMS teams of which seven recovered cardiac activity after immediate medical resuscitation. Overall, 106 patients (46.5%) received resuscitation by our mobile EMS teams. The Table 1 shows the demographic and clinical characteristics comparison of the resuscitated and not resuscitated groups.

**Table 1:** Shows the demographic and clinical characteristics comparison of the two groups

variables	All N=228	Resuscitation+ N=106	Resuscitation- N=122	p
Mean age ±SD	64±14	61±15	65±12	0.048
Men n (%)	134 (59)	68 (64)	66 (54)	0.42
Bystander CPR n (%)	18 (8)	16 (15)	2 (1)	0.02
Median No-flow time (min)	22 [10-34]	10 [3-17]	31[24-39]	<0.001
**Comorbidities** Diabetes n (%)	76 (33)	34 (32)	42 (34)	0.52
Hypertension n (%)	54 (24)	28 (26)	26 (21)	0.65
Coronary artery disease n (%)	42 (18)	20 (19)	22 (18)	0.9
Neoplasia n (%)	24 (10.5)	10 (9)	14 (11)	0.25
COPD n (%)	14 (6)	6 (6)	8 (6.5)	0.47
Stroke n (%)	14 (6)	5 (5)	9 (7)	0.11
**Initial electrocardiographic rhythm** Asystole n (%)	218 (96)	98 (92)	120 (98)	0.14
FV/TV n (%)	8 (3.5)	7 (7)	1 (1)	0.12
PEA n (%)	2 (0.5)	1 (1)	1 (1)	0.54

SD: Standard Deviation, CPR: cardiopulmonary resuscitation, COPD: Chronic Obstructive Pulmonary Disease, FV: Ventricular Fibrillation, TV: Ventricular Tachycardia, PEA: Pulseless Electric Activity

**Predictors of resuscitation:** in univariate analysis, three predictors were found to have significant association with the decision to resuscitate or not patients with OHCA as shown in Table 2. Multivariate analysis identified one independent predictor of resuscitation (adjusted OR; (95% CI); p): no flow duration (1.38 (1.24-3.55); p <0.001). The ROC curve determined a cut-off value at 18.5 min for the decision not to resuscitate; the area under the ROC curve was 0.976.

**Table 2:** Univariate analysis

factor	OR	[95% CI]	p
Age ≥ 65 years	1.2	[1.12-7.5]	0.045
Bystander CPR	1.35	[1.2-2.4]	0.02
No flow-duration	1.5	[1.3-2.6]	< 0.001

**Survival rate:** the overall out of hospital survival rate was 17% of resuscitated patients. Twenty two patients (21%) have recovered a cardiac activity, four of them had a second non recoverable cardiac arrest during the transport and 18 patients were transferred to the emergency departments. The survival time of these patients did not exceed 24 to 48 hours.

## Discussion

In this study, over on in two patients (3.5%) who sustained an out of hospital cardiac arrest, resuscitation is not attempted by mobile EMS teams as the chances of survival are judged as negligible by the time of assessment. These patients are characterized by a no flow duration over 18 min when no bystander CPR is attempted such that the cardiac rhythm has degenerated to asystole. It is well known that longer mobile EMS response intervals are associated with worse outcomes after cardiac arrest. Providers who experience a longer response interval because of initial distance from the scene, poor traffic conditions may have been less inclined to attempt resuscitation on arrival because of a pessimistic outlook or a higher likelihood of the patient meeting local criteria for obvious death [5]. The decision to begin resuscitation in a patient without vital signs is a complex one faced by mobile EMS care providers in a challenging setting that may include an uncontrolled setting, emotional family members and other bystanders. It is possible that some could be saved with bystander CPR provision. For that it is extremely important to train people to be able to act when faced with a CA, as undertaking CPR, until the emergency medical service arrives, can increase the victim's chances of survival [6]. Bystander CPR is a critical step in the chain of survival, increasing the chances that a victim will survive by two to four folds, which translates to one additional life for every 30 patients who receive bystander CPR [3,4]. As the majority of OHCAs occur in the home, bystander characteristics are more important than victim factors training communities to perform CPR can increase bystander CPR rates and overall survival [7,8]. Ventricular fibrillation (VF) and ventricular tachycardia without a pulse (VT), frequently, are the rhythms found in persons with witnessed CA, as a result of which it is extremely important that both CPR and defibrillation should be undertaken at an early stage [9,10].

The chance of survival reduces by 7.0%-10.0% with each minute of delay in defibrillation, and pulseless VF/VT can deteriorate to asystole as time passes, but undertaking CPR can prolong pulseless VF/VT, thus increasing the chances of successful defibrillation [11]. A systemic review of 67 studies found the proportion of patients in whom resuscitation is attempted varies between countries from 33% to 100% [4]. In all studies, the duration of no flow period was the major determinant of prognosis [12]. To obtain maximum survival chances a delay of less than 4 min for basic CPR, less than 8 min for defibrillation and less than 12 min for advanced CPR are recommended [12,13]. Improving the prognosis of OHCA according to the chain of survival criteria involves the education of the public to shorten the duration of the no flow. The best treatment is therefore based on rapid recognition, the quality of CPR and especially prevention [14]. In our study, 17% of the resuscitated population were recovered a sinus rhythm with pulse and transferred to the emergency department but no patient came out alive from the hospital. This rate of immediate survival was lower than that identified in other studies: 22% to 33% [13,15]. To improve the prognosis of OHCA many challenges must be faced by the citizen and the care system: greater involvement of the population in learning and practicing gestures of resuscitation; training ambulance dispatch staff to provide compression only CPR telephone instructions both reduces time to first compression and increases chances of bystander CPR initiation; broad but controlled dissemination of AEDs for the public; a more efficient organization of the pre hospital system both through its implication and by better coordination between the different actors.

**Study limitations:** our study has some limitations. First, the number of patients included as relatively limited to generalize the findings. Second, we did not independently verify the information reported by mobile EMS clinicians. It was therefore possible that other unmeasured factors may have influenced resuscitation decisions. Future studies should exploring in greater depth the reasons why resuscitation is withheld to better understanding of the decision making process and whether any other factors may influence decisions.

## Conclusion

The decision to resuscitate an out of hospital cardiac arrest depends more on the no flow time than other factors. Future studies should explore strengthening the chain of survival to increase the community bystander CPR response and evaluate the effect on the number of survivors from out of hospital cardiac arrest.

### What is known about this topic

- When cardiac arrest occurs, irreversible death will be within minutes unless circulation is restored;
- The mission of emergency medical service team is to save life. However, resuscitation attempts are not always appropriate. Characteristics of patients in whom resuscitation is withheld by ambulance staff are not well established.

### What this study adds

- This is the first Tunisian study that identifies potentially modifiable factors associated with the decision to resuscitate or not an out of hospital cardiac arrest.

## Competing interests

The authors declare no competing interests.
